# A case of successful maintained pregnancy after neoadjuvant chemotherapy plus radical surgery for stage IB3 cervical cancer diagnosed at 13 weeks

**DOI:** 10.1186/s12884-020-02895-y

**Published:** 2020-04-07

**Authors:** Ying Guo, Dandan Zhang, Yuhong Li, Yudong Wang

**Affiliations:** 1 The International Peace Maternity and Child Health Hospital, School of Medicine, Shanghai Jiao Tong University, Shanghai, China; 2 Shanghai Key Laboratory of Embryo Orignal Diseases, Shanghai, China

**Keywords:** Cervical cancer, Pregnancy, Stage IB3, Chemotherapy

## Abstract

**Background:**

Cervical cancer during pregnancy is rare. The management for stage IB3 during pregnancy remains unclear and challenging. We report a successful preserved pregnancy in a stage IB3 patient who was treated with neoadjuvant chemotherapy (NACT) plus radical surgery.

**Case presentation:**

A 36-year-old pregnant woman was diagnosed with a 5-cm-diameter stage IB3 squamous cell carcinoma of the uterine cervix at 13 gestational weeks. The patient received 5 courses of systemic chemotherapy with carboplatin and paclitaxel every 3 weeks, followed by caesarean section and radical hysterectomy. Both the mother and infant are in good general condition.

**Conclusion:**

This case suggests that NACT plus radical surgery could be one method to maintain pregnancy in stage IB3 cervical cancer diagnosed as early as 13 gestational weeks.

## Background

Cancer during pregnancy, the incidence of which is expected to rise, is defined as a tumor diagnosed in pregnant women or in the immediate postpartum [1, 2]. Cervical cancer is the second neoplasia diagnosed during pregnancy or postpartum, with an incidence arranging from 1.4 to 4.6 per 100,000 pregnancies [2]. The rarity of cervical cancer in pregnancy (CCIP) makes large-scale studies impossible. There are effective management guideline for CCIP which has been made recently [2]. The guidance is mainly based on case reports and experts’ opinions. Therefore, the management of CCIP remains a tough challenge.

We here report a detailed case of stage IB3 cervical cancer diagnosed at 13 gestational weeks successfully treated with neoadjuvant chemotherapy (NACT) using carboplatin and paclitaxel followed by radical surgery during pregnancy.

## Case presentation

A 36-year-old pregnant woman (gravida 3, para 1) presented at our gynecological oncology department with vaginal bleeding after thinprep cytologic test (TCT) at 13 gestational weeks. She reported no abdominal/pelvic pain and no medical and surgical histories. Gynecologic pelvic examination revealed a cervical lesion 5 cm in diameter without involvement of vagina and parametrium. An ultrasound scan revealed an enlarged uterus for a pregnancy at the 13th week. Gadolinium-free pelvic magnetic resonance imaging (MRI) confirmed that no regional lymph node engagement was documented. Squamous cell carcinoma of invasive non-keratinizing type was confirmed by cervical biopsy. Human papilloma virus (HPV) DNA testing was positive for HPV 18. The case was diagnosed as stage IB3 according to the latest 2018 International Federation of Gynecology and Obstetrics (FIGO) classification.

**Fig. 1:**
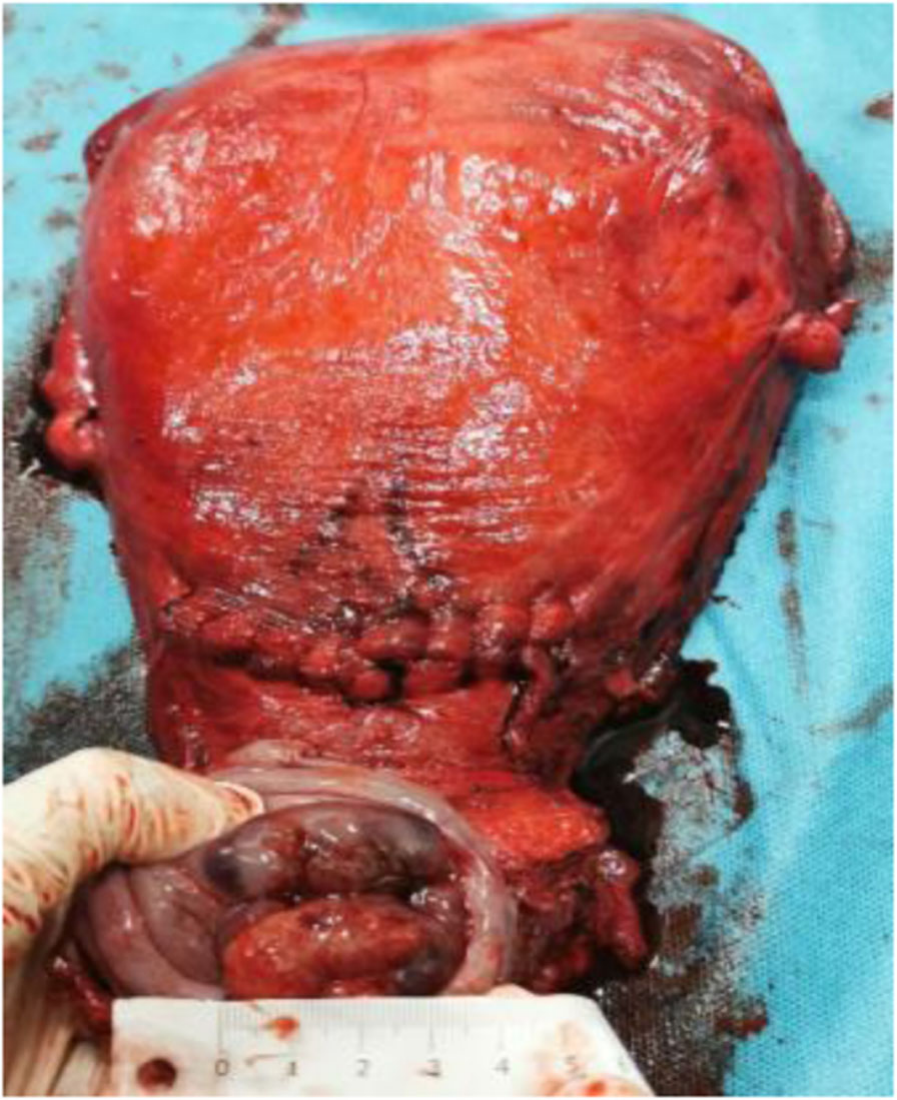
Surgical specimen of uterus and parametria after caesarean section and radical hysterectomy. The identifiable lesion was 3.5 cm in diameter which was measured by ruler

The patient strongly desired to maintain the pregnancy and refused to perform surgery. All the potential risks and complications of therapy were presented and the informed consent was signed. After thorough discussion in a multidisciplinary team (MDT) meeting, we decided for NACT with carboplatin (area under the curve of concentration × time [AUC]= 5 on day 1 every 21 days) and paclitaxel (175 mg/mq every 21 days), followed by caesarean section and radical hysterectomy with monitoring the evolution of the mass and pregnancy. The patient received 5 cycles of chemotherapy from 20 gestational weeks to 32 gestational weeks. The only toxic effects were slight nausea and vomiting. Concerning the advanced maternal age (36-year-old), prenatal screening for the common fetal autosomal aneuploidies was suggested. But malignancy among pregnant women could result in discordance between noninvasive prenatal testing (NIPT) results and the fetal karyotype [3]. Therefore, an amniocentesis was performed and revealed no chromosome anomalies at 24 gestational weeks. Fetal and maternal Doppler readings demonstrated no intrauterine growth restriction through pregnancy. After careful MDT discussion and a review of literature [4], fetal lung maturity was achieved for babies at 35 weeks and 3-week-interval between the last cycle of chemotherapy (32 weeks) and delivery was recommended [2]. Thus, a caesarean section at 35 weeks’ gestation was performed 3 weeks after the last cycle of chemotherapy to allow both maternal and fetal bone marrow to recover, followed by radical hysterectomy and pelvic lymphadenectomy. The caesarean section was performed under locoregional anesthesia, with conversion to general anesthesia for the hysterectomy and lymphadenectomy [2]. The infant was a female, with an Apgar score at 1 and 5 min of 9 and 10, weighing 2060 g (21th percentile according to WHO growth curves). After placental expulsion, radical hysterectomy plus pelvic lymphadenectomy were performed. As no lymph node engagement was indicated by MRI and assessment during the surgery, para-aortic lymph nodes dissection was not considered. The patient and infant were discharged on the twelfth postoperative day in good general condition. The identifiable lesion was 3.5 cm in diameter during the surgery (Fig. 1). Histologic report revealed a poorly differentiated cervical adenocarcinoma, locally adenosquamous carcinoma with 75% stromal invasion, invasion of the posterior vaginal wall, no lymphovascular space invasion, clear vaginal resection margins and negative pelvic lymph nodes. Postoperative radiotherapy was proposed. Extensive pathological examination of placenta and umbilical cord showed no metastasis of maternal malignancy. Neonate physical examination, blood count, biochemical analysis and auditory brain stem evoked potential test turned out to show no sign of abnormality. At last follow-up (4 months post-surgery) both the mother and infant are in good general condition.

## Discussion and conclusions

The current case suggests NACT using carboplatin and paclitaxel followed by radical surgery during pregnancy could be applied for the treatment of cervical cancer stage IB3 diagnosed in the first trimester of gestation for women who desire to preserve pregnancy. The case showed favorable outcome even with a delay of treatment by 22 weeks.

The management of cervical cancer depends on gestational age (first, second, and third trimester) at diagnosis, tumor stage and the patients’ wishes concerning the pregnancy. Guidelines has been provided which was based on a third international consensus meeting of members of the International Network on Cancer, Infertility and Pregnancy (INCIP) in collaboration and other international experts [2]. For pregnancy-preserving patients, therapeutic options include surgery with or without NACT and delayed treatment after delivery, while termination of pregnancy was suggested for women with advanced disease (stage IIB or higher or lymph node metastases). 70% of cervical cancers during pregnancy are diagnosed at stage I [5]. The management for stage IB3 cervical cancer during pregnancy is controversial, for which NACT or termination of pregnancy was recommended by INCIP if the diagnosis is made before 22 weeks’ gestation [2]. For the treatment of locally advanced disease (more than 4 cm in the diameter), the French guidelines propose termination of the pregnancy if the diagnosis is made before 18 weeks’ gestation, whereas the European consensus meeting guidelines propose NACT as the first option [5]. Seventy-six patients with IB1, IB2 and IB3 tumors after staging lymphadenectomy (mean 16 weeks of delay) presented excellent oncological outcome [5]. There was evidence that tumor response in pregnant women has been satisfactory with complete or partial response after NACT [6, 7]. The lesion in the present study was reduced after NACT, but the invasion of vaginal wall was revealed. Therefore, the efficiency of NACT remains unclear, which has only been investigated in a small number of trials. The optimal response (reduction of tumor volume more than 50%) rate after NACT in IB bulky tumours was 78.8% [8], while it was only 60.7% for tumours that exceeded 2 cm in the largest diameter [7]. The case in the present study exhibited good outcome with delay in treatment as long as 22 weeks, which brings new evidence for preserving pregnancy option for cervical cancer larger than 4 cm in the diameter diagnosed at 13 gestational weeks or even earlier.

Chemotherapy exposure after the first trimester showed comparable effects on the fetal malformations compared with the general population [9–15]. Generally, chemotherapy is feasible after 14 weeks of gestation, which is not suggested beyond 35 weeks because a 3-week-window between the last cycle of chemotherapy and delivery is of significance for the recovery of maternal and fetal marrow [2]. The prenatal platinum exposure has been associated with ototoxicity [16–21] and fetal growth restriction [22]. Up to now, only 3 cases were treated with carboplatin to the best of our knowledge [6, 23, 24]. No fetal and neonatal complications concerning carboplatin has been reported. Carboplatin has been reported as being the safest platinum derivative to be used in pregnancy [25]. In 2019 Zagouri performed a systematic review in which the chemotherapy was carried out ranging 18–30.6 weeks and indicated that taxanes administration during the 2nd and 3rd trimester of pregnancy is a safe choice for cervical cancer during pregnancy [7]. However, embryonal rhabdomyosarcoma (ERMS) has been observed in one 5-year-old child [26], which might be associated with the prenatal exposure to chemotherapy. As for the chemotherapy regimens of cervical cancer during pregnancy, paclitaxel/carboplatin weekly or 3-weekly was recommended according to the latest guideline of INCIP [2, 7]. The present case also suggests paclitaxel / carboplatin 3-weekly is safe for the offspring. However, longer follow-up is needed to define the safe outcome of the child.

It is suggested that delivery should be induced after 37 weeks to avoid neonatal morbidities and to assure the fetal maturity [2]. According to the literature, the pregnancies were mostly terminated by caesarean section ranging 31–38 weeks [1, 6]. Given the risks of vaginal delivery including tumor laceration, excessive bleeding, obstruction of the birth canal and implantation of malignant cells in perineum wound scar [27–29], caesarean section is indicated for cervical cancer [2].

In conclusion, this case adds a new case of stage IB3 cervical cancer in pregnant patient who maintains pregnancy by NACT plus radical surgery diagnosed at 13 gestational weeks or even earlier. By considering the literature, NACT using carboplatin and paclitaxel followed by radical surgery during pregnancy in pregnant patients appears to be safe and feasible for both the mother and infant. However, 4 months of follow-up is not enough in order to say that it is safe for the child and mother in the present case. Therefore, longer follow-up and large-scale studies are warranted in the future to shed new light on the maternal and fetal outcome.

## Data Availability

The data referred to in this case report was obtained from review of the patient’s medical record and is not publicly available.

## Abbreviations

NACT: Neoadjuvant chemotherapy
CCIP: Cervical cancer in pregnancy
INCIP: The International Network on Cancer, Infertility and Pregnancy
ERMS: Embryonal rhabdomyosarcoma
